# Supplementary material for: A Cluster Analysis of Risk Factors for Cancer across EU Countries: Health Policy Recommendations for Prevention
Source: Int J Environ Res Public Health. 2021 Jul 31;18(15):8142. doi: 10.3390/ijerph18158142 (PMC8346092; doi:10.3390/ijerph18158142)
Supplement: Supplementary file 1 [file ijerph-18-08142-s001.zip › ijerph-1292530-supplementary.pdf]

Supplementary materials

Table 1. Cluster analysis data by country.

ISO	Cluster	Cancer Ratio [per 100,000]	GDP/capita [EUR]	HC spending [%]	Years of edu	PM_{10}days	HPV segment	Alcohol [liters]	Smoke [%]	Diet [%]	Obese [%]	Sports Club [%]	Sports Activity [%]
BE	1	267.12	36,960.00	12.01	8.00	11.35	5.00	10.36	12.00	9.00	13.70	0.16	0.47
DE	1	291.61	37,090.00	14.15	8.50	8.76	3.00	11.99	9.00	2.00	16.40	0.24	0.48
DK	1	284.64	48,050.00	12.61	8.60	10.71	4.00	9.38	8.00	20.00	14.40	0.25	0.68
FI	1	217.54	38,570.00	12.71	7.50	8.07	5.00	8.51	4.00	10.00	17.80	0.12	0.66
IE	1	192.31	55,970.00	11.40	5.30	3.25	4.00	10.93	10.00	21.00	18.20	0.19	0.52
LU	1	213.19	91,440.00	13.60	4.60	4.75	4.00	11.83	5.00	14.00	15.10	0.21	0.54
NL	1	281.69	40,730.00	12.23	6.70	9.06	4.00	8.03	16.00	19.00	12.90	0.27	0.58
SE	1	236.31	46,350.00	12.43	9.20	9.12	5.00	7.16	1.00	30.00	13.40	0.22	0.70
BG	2	253.28	6360.00	11.28	4.20	63.63	2.00	11.30	31.00	24.00	14.40	0.02	0.11
CY	2	223.02	21,030.00	11.95	2.90	37.00	1.00	9.55	23.00	15.00	13.90	0.06	0.36
EL	2	274.24	16,380.00	10.75	4.60	40.67	2.00	10.56	29.00	29.00	16.90	0.05	0.31
HR	2	333.38	10,600.00	12.05	5.60	34.55	1.00	9.89	25.00	26.00	18.00	0.10	0.35
HU	2	346.91	11,400.00	11.91	4.70	28.52	5.00	10.90	22.00	3.00	20.60	0.05	0.38
LV	2	278.05	12,350.00	12.98	3.40	15.17	3.00	10.82	28.00	5.00	20.80	0.06	0.31
PL	2	279.95	11 190.00	13.09	4.40	55.30	2.00	10.48	21.00	3.00	16.70	0.03	0.28
RO	2	244.93	8 090.00	10.91	3.90	14.53	1.00	10.40	20.00	1.00	9.10	0.01	0.21
SK	2	240.50	14,710.00	12.78	5.40	22.22	1.00	10.78	27.00	4.00	15.90	0.07	0.34
ES	3	243.40	23,220.00	9.97	6.50	10.22	5.00	8.26	19.00	8.00	16.20	0.07	0.46
IT	3	293.26	27,260.00	10.19	6.70	33.27	4.00	7.14	11.00	6.00	10.50	0.07	0.30
MT	3	209.01	21,620.00	11.25	5.60	18.50	5.00	7.75	13.00	16.00	25.20	0.06	0.19
PT	3	263.10	17,350.00	9.11	5.90	9.87	5.00	10.54	6.00	18.00	16.10	0.04	0.28
AT	4	240.63	39,890.00	12.18	7.50	7.88	1.00	11.40	24.00	27.00	14.30	0.13	0.45
CZ	4	261.62	15,980.00	12.81	6.00	24.10	4.00	12.82	25.00	31.00	18.70	0.11	0.36
EE	4	258.72	15,820.00	13.90	4.80	4.43	1.00	16.64	26.00	17.00	19.70	0.12	0.39
FR	4	259.89	33,020.00	11.40	8.80	5.40	2.00	11.87	25.00	13.00	14.70	0.16	0.43
LT	4	269.23	12,850.00	13.08	4.30	18.87	1.00	14.42	18.00	11.00	16.60	0.08	0.37
SI	4	277.15	18,830.00	12.66	6.10	33.07	3.00	11.49	14.00	28.00	18.60	0.12	0.51

Source: own calculations

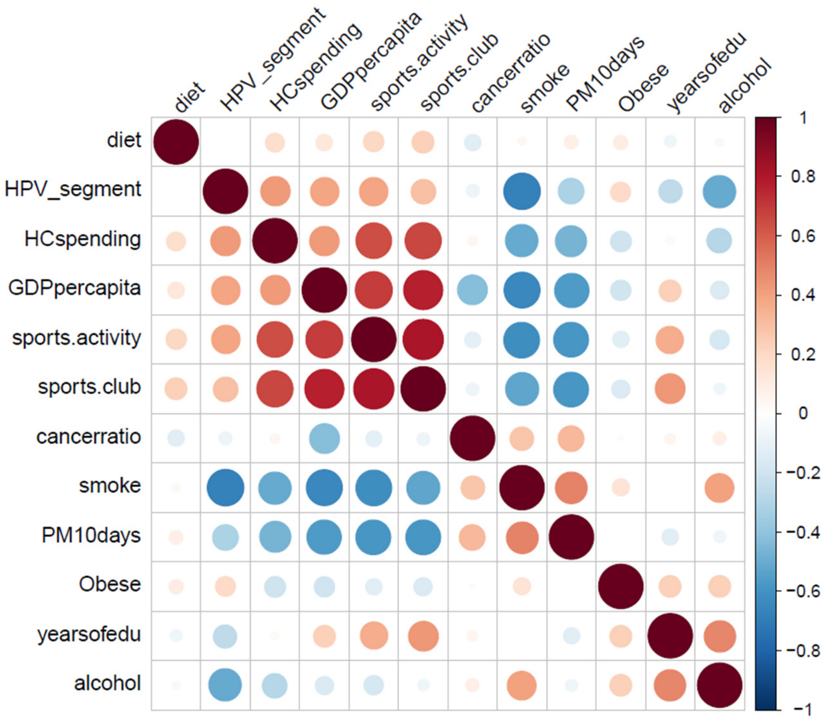

Figure S1. Correlation matrix of all variables. Source: own calculations

Table S2. Pearson correlation coefficients with p-values for all possible pairs of variables used in this analysis

Variable 1	Variable 2	Pearson coefficient (p-values)
alcohol	Obese	0.23
alcohol	PM10days	−0.07
alcohol	HPV_segment	−0.51**
alcohol	sports.activity	−0.18
alcohol	sports.club	−0.06
cancerratio	GDPpercapita	−0.43*
cancerratio	HC Expenditure	0.04
cancerratio	Years of Educ	0.05
cancerratio	Diet	−0.13

cancerratio	Smoke	0.27
cancerratio	Alcohol	0.08
cancerratio	Obese	0.02
cancerratio	PM10days	0.32
cancerratio	HPV_segment	−0.08
cancerratio	sports.activity	−0.12
cancerratio	sports.club	−0.08
diet	smoke	0.03
diet	alcohol	0.03
diet	Obese	0.1
diet	PM10days	0.09
diet	HPV_segment	−0.01
diet	sports.activity	0.2
diet	sports.club	0.23
GDPpercapita	HCspending	0.42*
GDPpercapita	yearsofedu	0.23
GDPpercapita	diet	0.13
GDPpercapita	smoke	−0.65***
GDPpercapita	alcohol	−0.16
GDPpercapita	Obese	−0.2
GDPpercapita	PM10days	−0.57**
GDPpercapita	HPV_segment	0.39*
GDPpercapita	sports.activity	0.69***
GDPpercapita	sports.club	0.77***
HCspending	yearsofedu	0.03
HCspending	diet	0.17
HCspending	smoke	−0.51**
HCspending	alcohol	−0.29
HCspending	Obese	−0.21
HCspending	PM10days	−0.47*
HCspending	HPV_segment	0.42*
HCspending	sports.activity	0.64***
HCspending	sports.club	0.66***
HPV_segment	sports.activity	0.39*
HPV_segment	sports.club	0.29
Obese	PM10days	0
Obese	HPV_segment	0.19

Obese	sports.activity	−0.13
Obese	sports.club	−0.16
PM10days	HPV_segment	−0.32
PM10days	sports.activity	−0.59**
PM10days	sports.club	−0.59**
smoke	alcohol	0.4*
smoke	Obese	0.14
smoke	PM10days	0.49**
smoke	HPV_segment	−0.67***
smoke	sports.activity	−0.62***
smoke	sports.club	−0.53**
sports.activity	sports.club	0.82***
yearsofedu	diet	−0.07
yearsofedu	smoke	0
yearsofedu	alcohol	0.48*
yearsofedu	Obese	0.23
yearsofedu	PM10days	−0.13
yearsofedu	HPV_segment	−0.26
yearsofedu	sports.activity	0.36
yearsofedu	sports.club	0.43*

Source: Authors’ own calculations. Note: Significance: *** <0.001, ** <0.01, * <0.05, <0.1.

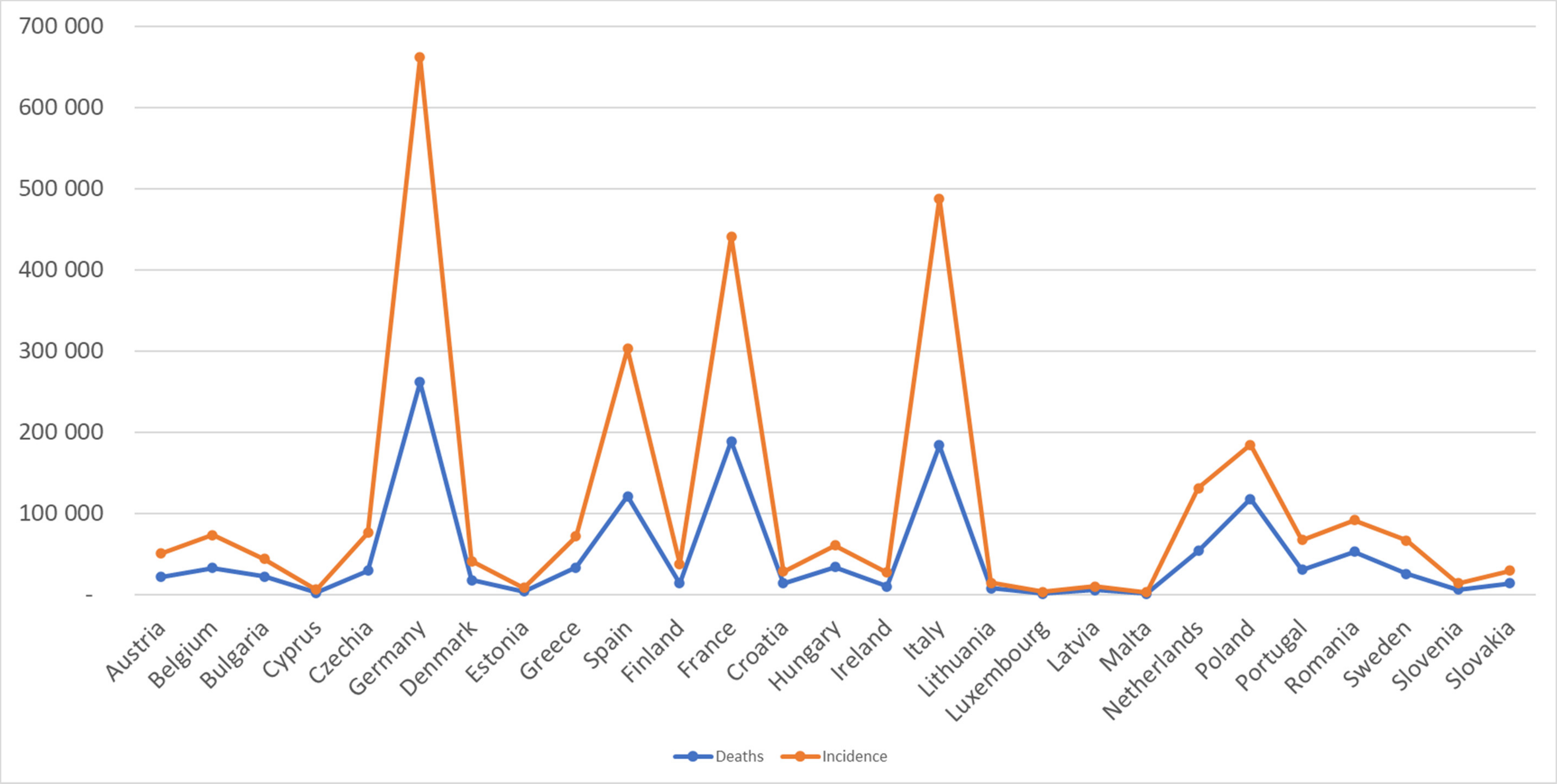

Figure S2. Cancer deaths and Cancer incidence per country, 2017. Source: authors' own calculations based on data obtained from the Institute for Health Metrics and Evaluation (IHME) [Dataset] Global Burden of Disease Collaborative Network. The Global Burden of Disease Study 2017 (GBD 2017) Cause-Specific Mortality and Incidence 2017.; Seattle, 2018. Note: Pearson correlation coefficient =0.99

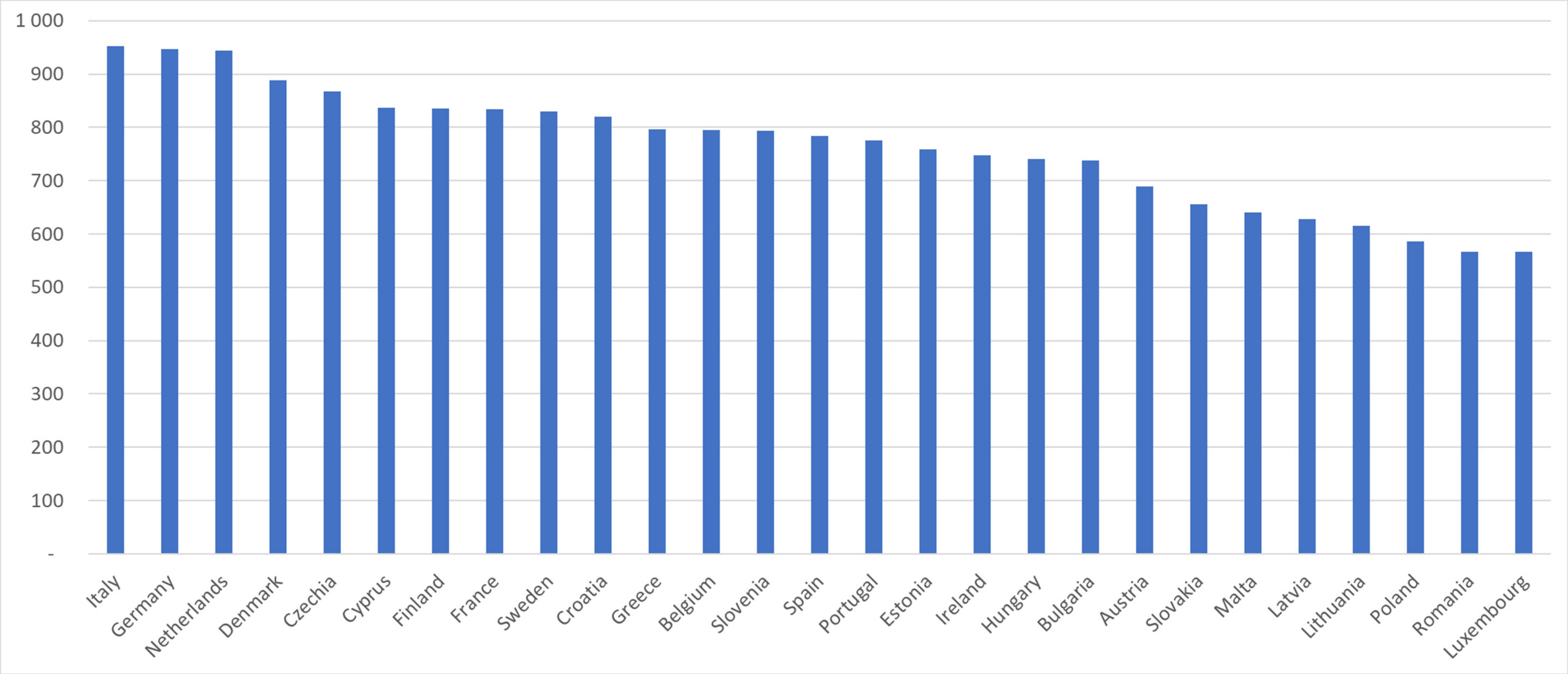

Figure S3. Cancer incidence per 100,000 inhabitants per country in 2017. Source: authors' own calculations based on Eurostat Population aged 18+ in 2017 (https://ec.europa.eu/eurostat/databrowser/product/view/DEMO_GIND) and data from Institute for Health Metrics and Evaluation (IHME) [Dataset] Global Burden of Disease Collaborative Network. Global Burden of Disease Study 2017 (GBD 2017) Cause-Specific Mortality and Incidence 2017.; Seattle, 2018.
